# Factors influencing the consumption of seafood among young children in Perth: a qualitative study

**DOI:** 10.1186/1471-2458-7-119

**Published:** 2007-06-25

**Authors:** Alexandra McManus, Sharyn K Burns, Peter A Howat, Lisa Cooper, Lynda Fielder

**Affiliations:** 1 Western Australian Centre for Health Promotion Research, Curtin University of Technology, Perth, Australia; 2 Centre for Behavioural Research in Cancer, Curtin University of Technology, Perth, Australia

## Abstract

**Background:**

This formative study sought to explore the factors that influence the consumption of fish and seafood among 4–6 year old children in the Perth metropolitan area. Focus groups were conducted with mothers of young children to gain insights into the enablers and barriers to regular seafood consumption in children, and the knowledge, attitudes and perceptions of their mothers to including seafood as a regular part of their children's diet.

**Methods:**

Purposive sampling techniques were used to select and recruit mothers of children aged between four and six years from within the Perth metropolitan area. A total of seven focus groups were conducted. Thematic content analysis was employed to code data generated and to extract major themes.

**Results:**

Findings indicated that all children of study participants had tried fish and seafood products, with some being exposed to a wide variety from an early age. Across focus groups, several dominant factors were apparent in influencing the frequency and type of seafood purchased and consumed. Perceived cost, freshness, availability/accessibility, and the level of confidence to prepare a meal to suit all family members were significant determinants of whether seafood featured regularly on the household menu. The influence of others in the family (particularly the husband or partner) also tended to impact upon the likelihood of serving fish and seafood, and the types of products mothers were willing to serve.

**Conclusion:**

Findings from this qualitative study indicate that interventions seeking to promote seafood (particularly fish) as an integral part of a healthy diet should address existing negative attitudes and beliefs around the storage and preparation of seafood. The influence of dominant male influences within the family unit should also be considered. Strategies directed at parents and children should include experimental 'hands-on' components to encourage experimentation, particularly focussing on ease of preparation and the variety of lower cost seafood available.

## Background

Establishing regular fish consumption as a healthy, cost effective option for families has the potential to impact upon their short and long-term health [1,2]. It also compliments the existing nutritional messages that relate to fruit and vegetables as essential to a healthy diet. Furthermore, the significant increase in cardiovascular diseases, overweight and obesity in childhood and adolescence facilitates the promotion of regular seafood consumption as a habitual part of a healthy diet throughout the lifespan [1,2].

Poor dietary choices increase the risk of chronic illness such as cardiovascular diseases (CVD), type 2 diabetes, overweight and obesity, and some cancers [2]. This is particularly important as it is estimated that 9 out of every 10 Australians have at least one risk factor for cardiovascular diseases (CVD), 3.5 million by cancers, up to two million are living with diabetes, and over 60% are considered overweight or obese [3,4]. Together these conditions account for the majority of the burden of disease in Australia with the highest prevalence being in lower socioeconomic areas (up to 2.3 times higher that high income areas) and in indigenous populations [5,6]. Research has shown that a healthy diet, that includes regular seafood consumption, is one of the major protective factors against these conditions [3]. There is also a growing body of evidence that specific types of seafood have a positive effect on conditions such as dementia [7], allergies [8], overweight and obesity [9], asthma [10], depression and bipolar disorders [11].

The purpose of this formative research was to explore the factors that influence the consumption of fish and seafood among 4–6 year old children in the Perth metropolitan area. This paper will summarise the findings from a series of focus groups and make recommendations for the promotion of seafood as an integral component of a healthy lifestyle.

## Methods

### Sampling

Purposive sampling techniques were used to select and recruit mothers with children aged between four and six years in the Perth metropolitan area. A combination of snowballing and volunteer recruitment methods were implemented through flyers, word-of-mouth and email distribution lists. Prospective respondents were identified and letters of invitation forwarded to them.

Prior to their involvement, participants were provided with an information sheet outlining the purpose of the research, the type of involvement required of them, who was conducting the research, that their participation was voluntary and their confidentiality was to be upheld. Active consent was obtained from participants prior to their involvement. A total of seven focus groups were conducted (n = 38 participants).

### Data Collection

Prior to the focus groups, participants completed a one page demographic questionnaire. This provided general information on personal characteristics of participants as well as indicating the number of children in their household. A focus group protocol was developed to provide information to focus group participants relating to the process and procedure of the group discussion. The protocol included: an introduction to the facilitator and observer; an outline of the purpose of the focus group; group rules relating to confidentiality, honesty, respecting others opinions; and clarifications of terminology to be used.

A focus group questionnaire was developed to provide facilitators with a series of questions related to fish and seafood consumption in young children. Guided by themes emerging from the literature together with concepts considered important to address the purpose of this formative study, predetermined areas of inquiry included: whether children had tried fish and what type; favourites type of fish; the type of fish usually purchased; how fish was prepared; issues around the mothers' experiences of serving fish as a family meal; and some of the barriers associated with fish becoming a regular family meal.

Whilst the focus group schedule was used to guide discussion based upon areas considered important to the purpose of the research, the flexibility of the discussion allowed the facilitator to follow valuable avenues of inquiry. Emergent themes provided direction for areas of further investigation during subsequent focus groups.

With permission from participants, each focus group was audio-taped for accuracy of transcription and analysis. A trained observer was present at all sessions to record the content of discussions. All focus group protocols were standardised were to ensure consistency in data collection whilst also allowing emerging themes to be followed during discussion.

### Data entry, management and analysis

Participants' responses to the demographic questionnaire were collated and responses expressed as proportions. Immediately following each focus group, responses to the focus group questions were transcribed and analysed thematically. [5-7] The data from each focus group were then amalgamated and the major themes detailed using quotes from participants to support these findings.

### Ethical considerations

Ethics approval was received from the Curtin University Human Ethics Committee to conduct this research. This complies with the Helsinki Declaration for research conducted with humans. All participants provided written consent prior to being involved in this study.

### Limitations

It should be noted that this study was exploratory and descriptive in nature thus cannot be extrapolated beyond the study participants. As such, the findings of this qualitative study provide some direction for further research around the issues raised. Furthermore, the study sample was also taken from within the Perth metropolitan area where seafood supplies are readily available. Larger studies are required to provide empirical evidence as to whether these findings are indicative of the target population as a whole.

## Results

A total of seven focus groups were conducted (n = 38 participants) with women aged between 23 and 45 years representing 44 children, from a mid-level socio-economic status and a wide variety of ethnic backgrounds. A criterion for inclusion into the focus groups was that participants were mothers of young children. Several participants also had children outside the age group of interest however their responses were confined to information about family experiences of children within the target group. Focus groups results are presented thematically.

Results presented within this section are based upon the responses from the 38 focus group participants to discussion points (underlined), with direct quotations recorded in *italics*. All numbers in brackets (n = x) refer to the proportion of participants out of a possible 38.

### Have your children tried fish or seafood before?

Participants from all focus groups indicated they and their children had consumed fish or seafood. The discussions centred around the introduction of different types of seafood and fish depending upon what was being prepared for the family. Most participants indicated that they prepared one family meal rather than a separate meal for children and adults.

### What types of fish or seafood have your children tried?

The types of seafood children had tried were relatively diverse. Most children had tried tuna (n = 35), prawns (n = 33), fish and chips (n = 34), fish fillets (n = 23) and white flesh fish (n = 21).

'... we often get just like a firm white fillet and sort of crumb it ourselves'

'... homemade fish and chips using frozen fish'

'She (child) has always loved tinned tuna right from very early solids eating'

Some had either not been exposed to crustaceans or had expressed negative attitudes to these species for a variety of reasons.

'They sort of turn their nose up at things like mussels, crabs and anything in a shell'

'They haven't really gone into the more exotic seafood'

'They're too scared to actually eat the crab because they see them when they're alive'

### What is your child's favourite type of fish or seafood?

The favourite types of fish or seafood noted were tuna (n = 31), fish and chips (n = 25), prawns (n = 12), and boneless, white flesh fillets (n = 10). With regards to fish and seafood meals that children did not like, an emerging theme was that young children tended to prefer dishes where they could see the fish meat clearly rather than those where the fish was hidden in other ingredients such as mornays or in sauces.

'They don't like it prepared in any sort of sauces, they don't like fish mornay or anything like that'

'Her favourite is prawns but I think that might just be the scarcity value because we don't have them very often, they're a treat'

'Her favourite breakfast is tuna and pickles'

### What type of fish or seafood do you usually purchase?

When asked about the type of fish usually purchased for their family, participants in the majority of focus groups said this decision was influenced primarily by the price (n = 34) and the freshness (n = 32) of the product. Boneless fillets, tinned tuna and frozen products (n = 27) were common purchases among participants.

'We don't eat probably as much as what we should do, because of the price'

'I won't go over $20 a kilo'

'I always in the pantry have tins of sardines and tuna'

'I must admit in my house I would very rarely buy fresh fish'

'I'll never buy frozen fish I'll only buy the fresh fish but I will freeze it in my own freezer, at least then I know when it was frozen'

Trends in where fish and seafood products were purchased by participants was also apparent, with most participants preferring to purchase products from a specific supermarket chain, seafood specialty outlets or fish mongers. However, accessibility to these outlets was a perceived barrier among participants.

### How do you usually prepare fish to be served?

The preparation and cooking style of fish or seafood most often included crumbed (n = 28), oven baked (n = 23), pan fried (n = 22), barbequed (n = 19) and battered fish or seafood (n = 17). Themes relating to cultural influences on the methods of preparation and how fish was served as a family meal also emerged from several focus groups. Across focus groups, almost half of the participants said they lacked confidence when it came to preparing and cooking fish (n = 18) and tended to remain with dishes they were comfortable cooking and they knew the family liked.

'We used to do a lot more fish baking (whole fish) but just didn't want to have to deal with the bones, I was just too nervous of all that so it was kind of dropped off the menu'

'I need more creative ways to prepare it and a cookbook which has got simple, tasty ways of cooking fish'

'... if I cook fresh fish, because it is quite expensive then I'll just concentrate on the taste of it'

'Barbequed because then my husband goes outside and cooks it'

'I'm still I guess inexperienced in cooking fish in different ways, like if I know how to cook it one way I'll always cook it that way because I know it won't be dry or mush'

'It's a man's job in our house. It's always been a man's job in Turkey, ... men clean the fish and women clean the kitchen afterwards'

'In France you would have salad rather than vegetables with fish'

### What are your experiences when serving fish/seafood as a family meal?

Several commonalities were found between participants regarding their experiences when serving fish or seafood as a family meal. The majority of participants considered bones to be an important factor when serving fish to their children.

'I have no problems with the bones because I'm Asian we usually use chopsticks you know, but my problem is with the children and I'm constantly like did I clean enough or did I check enough'

'Mine would just eat them (bones) .... they eat everything, so that's not such a pain for me'

'We usually try to de-bone it as much as possible before hand but if it is fish that has bones in it it'll be like who can find the most bones, it kind of becomes a competition'

The influence of personal attitudes towards fish and seafood (n = 33), and those of children or husbands/partners also impacted upon the type and frequency of fish served as a family meal (n = 27).

'My husband doesn't really like fish so I find it kind of hard'

'I find what I like ... I'm quite fussy so they don't probably get to taste that much'

'My kids are just reluctant to try new things. You have to disguise it. I remember the first time they ate salmon I had to tell them it was Barbie chicken because it was pink'

'Our little boy won't touch like the prawns and the octopus, the squid rings and that could partly be because I don't like it you know, I like white fish with no bones and he tends to be the same'

'We go through stages where we would have fish on a weekly basis and then my husband would say look I'm really sick of fish lets go off it so wouldn't have it for maybe three or four months'

A few participants (n = 8) were willing to serve separate seafood or fish-based meals for children that were less spicy than those prepared for adults. This allowed adults to enjoy fish and seafood dishes considered unsuitable for children.

'For the children I tend to crumb it, I dip it in egg and flour and then crumb it and that looks like nuggets you know'

'I must admit we will split it up sometimes, if we really fancy a curry and they're not going to eat that then we will feed them earlier, we actually quite enjoy splitting up meals'

Strategies employed to introduce and encourage the ongoing consumption of a variety of fish and seafood meals by children included eating fish before being permitted to eat chips or calling fish by a similar food that the children had experienced and enjoyed (e.g. chicken).

'I find the gimmicky thing for young children worked really well'

'We use chips as an incentive to get the kids to eat fish'

'The only reason they eat fish is because of the association with chips'

'If I have my nephew over we call it chicken'

'For a long time everything was chicken, as long as it was called chicken she ate it'

### What do you think would encourage your family to eat more fish?

Price emerged as the dominant factor influencing the consumption of fish or seafood (n = 32). Availability and accessibility to good quality seafood outlets (n = 27), freshness of the products available (n = 25) and the availability of good quality boneless varieties (n = 21) were also considered important determinants of the frequency of fish consumption.

'... main thing for me is availability, trying to find a good fish monger'

'We only go for fresh fish that we can buy, we won't buy frozen fish'

'I find that I am buying more fish now because meat has got so expensive, so there's not such a huge difference in the price between meat and fish'

'I still think that parents have control over what the kids eat and you still have to encourage adults to eat more fish before you can encourage the kids to eat more'

### What do you think are some of the barriers to fish becoming a regular family meal in your community, that is including fish in meals two to three times per week?

When asked to discuss perceived barriers relating to fish becoming a regular family meal in their community, several dominant themes were evident. Participants considered the price of fish and seafood products to be the main barrier for regular consumption (n = 33). Lack of preparation and cooking skills (n = 20), availability and accessibility of high quality products (n = 18), whether family members like fish or seafood (n = 14), availability of (boneless) filleted fish (n = 12), and the smell associated with fish (n = 12) all featured prominently during discussions.

'... it's hard to ruin chicken, you can't really ruin a lamb chop but you can ruin fish very easily'

'I try to limit it to fifteen (dollars), otherwise I don't buy it, we don't eat fish'

If you do like a weekly shop when I buy fish I like to cook it that night so I don't usually buy a lot of it'

'... there is a bit of a fine art to cooking seafood and maybe people are afraid to risk the money that it costs you know to experiment'

## Discussion

Of the research that has been published, emerging themes surrounding the frequency and type of seafood served as a meal seem to be strongly determined by the presence and the age of children in the household. Several studies have reported that the presence of children may lead to some resistance towards seafood consumption [12,13]. Further, the development of negative perceptions associated with seafood, including smell during preparation, taste and 'the family do not like seafood' seem to be greater when teenagers are present as opposed to younger children [12]. It has been reported that the family 'norm', regarded as 'the family don't like seafood' increased with increasing household size, a trend which was mainly associated with the presence of children over the age of eight years [13]. Furthermore, teenagers under the age of 18 may have an indirect negative impact on seafood consumption through their perception of fish, resulting in significantly lower consumption frequency of fish products for family meals. Although teenagers were not the focus of this study, the findings clearly supported current literature that children had a strong influence on the type of foods prepared and consumed by families.

When investigating trends in the type of seafood consumed, households with young children more frequently eat processed fish, which is often not associated with poor taste, bad smell and variable quality and supply [12]. Highly processed fish products, including battered, crumbed and meals in sauces are regarded as convenient, easy to cook and popular with the family [14]. Processed seafood is also the only type of seafood for which the question of whether the family likes seafood appears to have no effect on consumption levels [12]. The findings of this study support these findings with most participants indicating a preference for processed seafood products often with accompanying high fat food (e.g. fried chips). Given the potential health benefits of regular seafood consumption, it is important to ensure that promotional health messages include healthy ways to prepare and consume seafood and that they do not include high fat accompaniments.

Reports suggest that consumers view processed varieties as subtle and meaty, with coatings and sauces making them colourful, appetising and may mimic traditional home cooking. Such products tend to disguise the qualities of fish and seafood that do not appeal to many consumers, especially children. Fresh fish on the other hand, which tends to generate both the strongest negative and positive beliefs, is often perceived as expensive, difficult to buy, prepare and cook, and has unpleasant physical properties [12,13]. Such factors impact strongly upon decisions to serve fish and seafood as a family meal. If the family does not want fish on the menu, arguments for eating fish (such as health benefits) are not considered, resulting in low fish and seafood consumption. An underlying finding in the present study indicated that seafood (particularly fish) was not presented to the children of participants again if they did like or eat the first seafood-based meal presented to them. This finding should be further explored given the varying tastes, textures and modes of preparation of seafood available.

When considering factors that facilitate the consumption of fish and seafood in households with young children, the interaction between social norms and moral obligations are important constructs to consider. Social norms from other family members (husband and children) have been shown to impose a significant negative impact on the frequency of seafood consumption (clearly evident in the present study). However, this pressure can be lessened through the presence of moral obligations to perform the behaviour for other reasons, such as the provision of a healthy meal for the family. The co-existence of both social pressure to adhere to family attitudes and preferences, alongside moral obligations to be responsible for the family's health may both influence the frequency and type of seafood products consumed [15].

Some of the barriers mentioned above to encouraging regular fish and seafood consumption, particularly in children, can be addressed through targeted efforts towards parents, school and the children themselves. Negative attitudes towards the purchase, preparation and taste of seafood of the adults in this study influenced what was offered to their children. Resistance against the consumption of fish may be reduced by developing improved products that better suit the tastes and preferences of younger age groups, along with the promotion of taste advantages and variety of preparation options available with high quality seafood products. Particularly attention should be placed on taste which is one of the main drivers behind the consumption of most foods, including seafood [13].

## Conclusion

This formative study sought to identify and examine some of the issues surrounding barriers and enablers to regular seafood consumption in young children and the knowledge, attitudes and perceptions of mothers to including seafood as a regular part of their children's diet.

Focus group results indicated that most children had tried a variety of seafood. The main barriers to regular consumption of seafood (including fish) identified by participants were cost and lack of knowledge of how to choose, prepare and store fresh seafood. Most participants indicated quick, wholesome and easy to prepare recipes for seafood were difficult to find. One interesting barrier that emerged was the dominance of the father or adult male preferences in the meals prepared. If the dominant male in the household did not eat seafood, then it was rarely prepared.

Foods regularly eaten as children became dominant foods prepared and eaten in adulthood. Parental influences and childhood experiences had an enormous influence on the foods eaten and prepared by participants in all focus groups.

## Recommendations for future research

The following are recommendations for future research based on the findings from this descriptive study.

1. The influence of fathers and male carers in the foods purchased and prepared in households should be investigated to identify ways their influence could be used to support the regular consumption of seafood as part of a healthy diet.

2. Further research should be conducted to investigate if guides to the selection and storage of good quality seafood are required.

3. Quick 'foolproof' recipes and hints of preparation of seafood should be developed to ensure that seafood is not 'wasted' through inappropriate preparation or high fat accompaniments.

4. The cost and availability of seafood were perceived barriers to regular consumption. Industry should be lobbied to provide and promote lower cost varieties of seafood that are readily available (including suggestions of ways to prepare them).

5. The effectiveness of strategies directed at parents and carers to overcome negative perceptions retained from childhood around seafood consumption and preparation should be investigated.

6. Strategies directed towards young children centring around experiential examples with 'hands on' preparation and cooking (were appropriate) may be effective. Future interventions that include an educational component directed at children and families should include practical sessions where preparation and tasting of a variety of seafood is an integral program component.

## Competing interests

The author(s) declare that they have no completing interests.

## Authors' contributions

AM conducted research, analysed data, prepared report, PH & SB provided support to the development of the research proposal, LC & LF conducted literature review. All authors read and approved the final manuscript.

## Pre-publication history

The pre-publication history for this paper can be accessed here:


